# Likelihood of secondary surgery due to osteoarthritis following ankle fracture fixation: a systematic review

**DOI:** 10.1186/s13018-026-06928-8

**Published:** 2026-05-13

**Authors:** Rebecca Beni, Adrian Kendal, Diego Agustín Abelleyra Lastoria, Conor Hennessy, Simon Abram, James Masters, Matthew L. Costa

**Affiliations:** 1 Nuffield Department of Orthopaedics, Rheumatology and Musculoskeletal Sciences, University of Oxford, Oxford, UK; 2 St. George’s University London, London, SW17 0RE UK

**Keywords:** Ankle fracture, Post-traumatic osteoarthritis, Ankle fusion, Ankle replacement, Reoperation, Systematic review

## Abstract

**Background:**

Ankle fractures are common injuries that can lead to long-term complications, including post-traumatic osteoarthritis. The rate of surgical intervention for osteoarthritis following fracture fixation is unclear. Understanding the burden of subsequent procedures is essential to inform patients and guide treatment strategies. This review investigates the risk of surgical interventions for osteoarthritis after surgical fixation of ankle fractures.

**Methods:**

We performed a systematic review following PRISMA 2020 and registered the protocol in PROSPERO (CRD42024615284). We included studies of adults (≥ 16 years) with acute ankle fractures treated surgically who later required operative treatment for osteoarthritis. Two reviewers independently performed data extraction and risk-of-bias assessment (Newcastle–Ottawa Scale). Because of clinical and methodological heterogeneity, we synthesised findings narratively.

**Results:**

From 2,425 records screened, eight cohort studies (n = 133,367) met inclusion criteria. The reported risk of ankle fusion or replacement due to osteoarthritis ranged from 0 to 4.2%, with mean follow-up ranging from 17 months to 21 years. One large registry study contributed the majority of patients.

**Conclusions:**

Reported rates of re-intervention for post-traumatic ankle osteoarthritis are low in the short to medium term but interpretation is limited by heterogeneity, generally short follow-up, and reliance on a single large registry cohort. High-quality, long-term studies are needed to determine the true lifetime burden and modifiable risk factors.

**Supplementary Information:**

The online version contains supplementary material available at 10.1186/s13018-026-06928-8.

## Background

Ankle fractures are common, with an incidence of almost 100 per 100,000 people in the UK in 2024 [1]. Patients younger than 40 years comprise almost a third of cases [2]. Management of these injuries is dictated by the stability of the mortise joint [3]. If this is stable, analgesia, splinting and weight-bearing as tolerated are the mainstay treatments. Early internal fixation is indicated in most patients younger than 60 years of age with an unstable joint [3], whereas close contact casting is indicated for those aged over 60 years [4].

Ankle osteoarthritis (OA) is a significant long-term consequence of ankle fractures [5]. Notably, 60–80% of ankle osteoarthritis cases are post-traumatic, in stark contrast to hip and knee osteoarthritis, where trauma accounts for only 10% of cases [5, 6]. A meta-analysis found the 1-year rate of radiographic arthrosis post ankle fracture varied between 25 and 34%, associated with injury severity [7]. However, radiographic findings do not always correlate with symptoms, and multiple factors influence the decision for surgical intervention [7]. Salvage surgery for severe arthritis of the ankle usually involves either ankle fusion or replacement; outcomes are comparable, but each intervention has its own limitations and complication profile.

The long-term burden of secondary surgery attributable to osteoarthritis remains unclear following ankle fracture fixation, with no previous systematic reviews performed. This lack of evidence prevents clinicians from adequately informing patients of the potential outcome of their injury and the risk of needing subsequent surgery due to arthritis. The primary outcome of this systematic review was to identify studies reporting further surgeries due to ankle arthritis following ankle fracture fixation.

## Methods

This systematic review was conducted in accordance with the PRISMA 2020 checklist [8]. We prospectively registered our review in PROSPERO (Registration number: CRD42024615284).

### Population

We included studies if they involved patients ≥ 16 years old with an acute ankle fracture which was treated surgically, and who subsequently required further surgical intervention for post-traumatic osteoarthritis.

We excluded studies involving patients younger than 16 years of age, Pilon or talus fractures, ankle injuries not involving a fracture, studies reporting outcomes in patients with congenital or developmental abnormalities of the ankle joint, fractures managed by amputation, and conditions not related to acute trauma. We also excluded patients with pre-existing ankle osteoarthritis or systemic conditions affecting joint health (e.g., rheumatoid arthritis, osteoarthritis due to other causes, or metabolic bone diseases).

### Search strategy and data extraction

A database search was conducted via OVID on Embase and Medline on 02/05/2025. The search, assessment of eligibility and data extraction were performed independently by two reviewers (RB, DAAL). The search strategy is provided in Appendix 1.

Cross-sectional studies, cohort studies, case–control studies, case series, and randomised controlled trials were eligible. Systematic or literature reviews, case reports, letters to the editor, and cadaveric studies were not eligible.

Baseline patient characteristics at the time of their ankle fracture (including patient age and gender, BMI, ethnicity and comorbidities) and study design (sample size, setting, study design and follow-up duration) were extracted from each study. Additional data included fracture type and classification, mechanism of injury, imaging modalities, treatment details (type and timing of surgery), complications, type and timing of surgical interventions for osteoarthritis (OA), and any further surgeries reported. Data extraction was conducted independently by two reviewers. A narrative synthesis was performed due to substantial heterogeneity in study designs, patient populations, fracture patterns, treatment approaches, and reported outcomes, which precluded meta-analysis.

The primary outcome was the risk of surgical intervention after the index fracture of the ankle where the indication for surgery is osteoarthritis. The covariates collected included patient-specific characteristics such as age, sex, and BMI, fracture type, treatment modality, post-operative complications, time to treatment, and other clinically relevant variables.

### Methodological appraisal

The level of evidence and risk of bias of each included study were evaluated independently by two reviewers (RB, DAAL). Each study was appraised with a tool reflecting the study design. The quality assessment of the included research was conducted using the Newcastle–Ottawa Scale (NOS) for assessing the quality of nonrandomised studies [9].

## Results

**Fig. 1:**
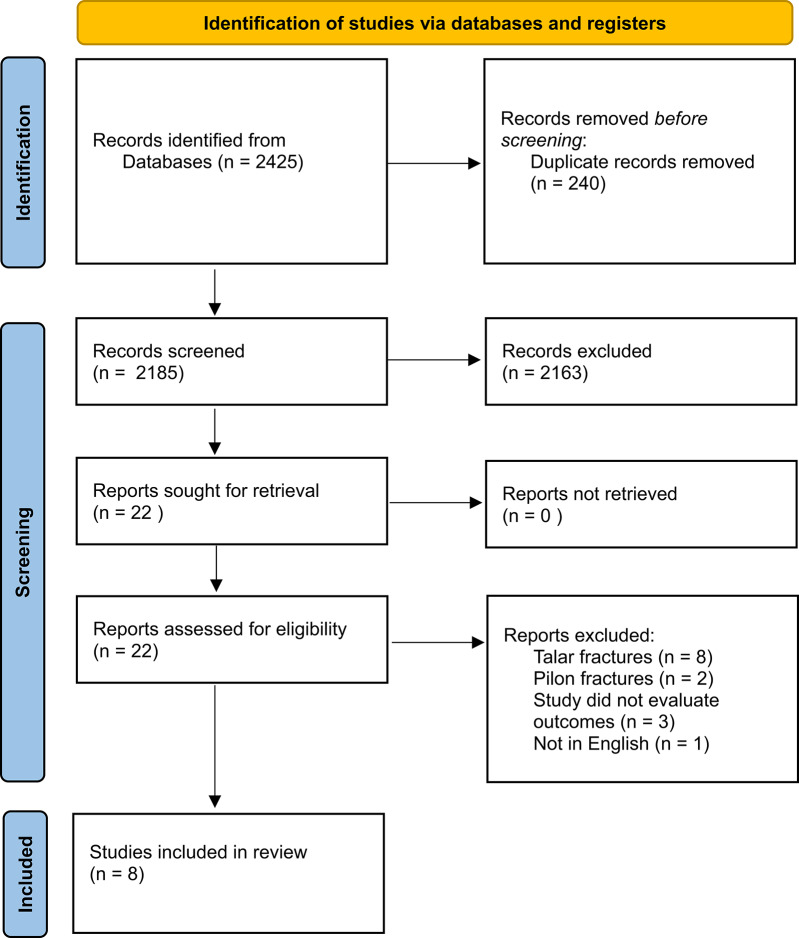
PRISMA diagram showing the study collection process

A total of 2,425 records were screened, with 22 potentially eligible studies undergoing full-text screening. Fourteen were excluded based on the eligibility criteria. Eight studies were included, evaluating 133,367 patients. The majority of these patients were reported in the study by Axelrod et al. [10], which included 44,133 operative ankle fixation (OAF) and 88,266 non-operative ankle fixation (NOAF) cases. The remaining included studies contributed to a total of 968 patients. Five studies were retrospective cohort studies and three were prospective cohort studies, one of which was a registry study. Five studies were based in Europe, two in America, and one in Asia (Fig. 1, Table 1 and 2).

**Table 1:** Baseline study data of the included studies

References	Country	Study design	Sample size	Setting
Kohake et al. [12]	Germany	Retrospective cohort study	61 patients (21 with syndesmotic rupture, 40 without)	University hospital in Germany (Medical School Hanover)
Verhage et al. [13]	Netherlands	Retrospective cohort study	169 patients	Level 1 trauma hospital (Haaglanden Medical Centre, Netherlands)
Lambers et al. [14]	Netherlands	Prospective cohort study	50 patients	Academic Medical Centre Amsterdam
Regan et al. [15]	USA	Prospective cohort study	141 patients	Academic medical centre with 2 Level 1 trauma centres and a tertiary care centre
Ray et al. [17]	UK (Scotland)	Retrospective cohort study	120 patients	University-affiliated trauma centre
Chou et al. [16]	Taiwan	Retrospective cohort study	49 patients (27 ORIF, 22 arthroscopically assisted reduction and minimally invasive surgery (AARMIS))	Level 1 trauma centre (Taipei Veterans General Hospital)
Macera et al. [11]	Spain	Retrospective cohort study	378 patients	University-affiliated hospital
Axelrod et al. [10]	Canada	Retrospective cohort study	44,133 operative ankle fixation (OAF), 88,266 Non-operative ankle fixation (NOAF)	Data collected via Ontario Health Insurance Plan (OHIP) Physicians Billings Database, ICES Physician Database, which captures demographic and training information about Ontario physicians; and the Canadian Institute for Health Information (CIHI) Discharge Abstract Database (DAD) and Same Day Surgery (SDS) databases

**Table 2:** Baseline demographic data of the included studies

References	Age	Gender	BMI	Ethnicity	Comorbidities
Kohake et al. [12]	Mean: 59 years (screw group), 54 years (no screw group)	11 males (52%), 10 females (48%) (screw group); 21 males (53%), 19 females (47%) (no screw)	Mean BMI: 28 (screw group), 26 (no screw group)	Not recorded	Not recorded
Verhage et al. [13]	Mean: 52.3 years	67 males (39.6%), 102 females (60.4%)	Mean BMI: 27.6	Not recorded	Diabetes: 7.1%
Lambers et al. [14]	Median: 36 years / 58 years (16–84)	34 males (68%), 16 females (32%)	Not recorded	Not recorded	Not recorded
Regan et al. [15]	Mean: 46 years	69 male patients (49%) and 72 female patients (51%)	Not recorded	Not recorded	Not recorded
Ray et al. [17]	Mean 42.7 years (16–85)	65 males (54.2%), 55 females (45.8%)	Not recorded	Not recorded	Not recorded
Chou et al. [16]	ORIF: 53.3 ± 15.8 years AARMIS: 50.9 ± 15.9 years	ORIF: 11.1% male, 88.9% female AARMIS: 22.7% male, 77.3% female	Not recorded	Not recorded	Diabetes: ORIF = 7.4%, AARMIS = 9.1%
Macera et al. [11]	Mean: 47.2 years	264 patients were males (69.8%) and 114 females (30.2%)	Mean BMI: 22.6 (18.5–35.4)	Not recorded	Not recorded
Axelrod et al. [10]	Mean 48.49 ± 18.20	Female 41.6%, male 58.4%	Not recorded	Not recorded	Diabetes: NOAF 11%, OAF 10.4% HTN: NOAF 26.8%, OAF 27.4% Fragility: NOAF 10.7%, OAF 19.3%

Participants were managed predominantly in academic or level 1 trauma centres. Mean patient age in the studies ranged from 36 to 58 years. Fifty one percent of the included patients were male. Mean BMI was reported in only three studies [11–13].

The fracture types varied between studies (Table 3). These were classified using the Weber (n = 2), Lauge-Hansen (n = 3), AO Foundation/Orthopaedic Trauma Association (AO/OTA) (n = 5) classifications. One study relied on register coding for fracture classification. The mechanism of injury was specified in three of the eight included studies [14–16]. Lambers et al. [14] reported injuries resulting from falls (44%), motor vehicle accidents (8%), and sports-related incidents (48%) in patients with Maisonneuve-type pronation–external rotation fractures specifically. All studies included open reduction and internal fixation as the surgical treatment. Chout et al. [16] also used arthroscopically assisted reduction and minimally invasive surgery in half of the cases. Follow-up duration varied considerably across the included studies, ranging from a minimum of 17 months to 21 years (median = 51 months, interquartile range 93.7 months).

**Table 3:** Characteristics of the included studies

References	Type of ankle fracture	Classification	Mechanism of injury	Fracture confirmation	Type of treatment
Kohake et al. [12]	Weber B-type ankle fractures	Weber	Not reported	Weight-bearing plain x-ray	ORIF; syndesmotic screw fixation if needed
Verhage et al. [13]	Trimalleolar fractures with posterior malleolar involvement	Lauge-Hansen and AO Foundation/Orthopaedic Trauma Association (AO/OTA)	Not reported	Preoperative lateral X-rays, postoperative X-rays, CT scans	ORIF; syndesmotic screw if required
Lambers et al. [14]	Maisonneuve-type pronation-external rotation ankle fractures treated with syndesmotic screws	AO/OTA	Falls (44%), motor vehicle (8%), sports (48%)	X-ray	ORIF with syndesmotic screws
Regan et al. [15]	Unstable ankle fractures	Lauge-Hansen and AO/OTA	100 low-energy twisting while falling (71%), 13 motor vehicle collisions (9%), 11 pedestrian motor vehicle accidents (8%), 8 sports-related injuries (6%), and 9 other mechanisms of injury (6%)	X-ray	ORIF with plates and screws
Ray et al. [17]	Various (classified using Lauge-Hansen & AO)	Lauge-Hansen and AO/OTA	Not reported	X-ray (AP, lateral, mortise views)	Syndesmotic fixation
Chou et al. [16]	Trimalleolar fractures	AO/OTA	Sprain: ORIF (25.9%), AARMIS (22.7%) Fall: ORIF (37.0%), AARMIS (50.0%) Motor Vehicle Accident: ORIF (37.0%), AARMIS (27.3%)	X-ray, CT scans	ORIF vs. AARMIS
Macera et al. [11]	Unstable ankle fractures	Danis–Weber classification	Not reported	X-ray	ORIF
Axelrod et al. [10]	Single or multiple malleolar fractures	Register coding	Not reported	Register coding	ORIF

### Study quality assessment

The results of the quality assessment are shown in Table 4. Two studies carried a high risk of bias [10, 16], three were deemed to carry moderate risk of bias [12, 13, 17], and three were at low risk of bias [11, 14, 15]. Common limitations across studies included unclear exclusion of patients with pre-existing osteoarthritis [10–17] and incomplete follow-up data [12–15, 17].

**Table 4:** Risk of bias assessment of included studies

References	Representativeness of the exposed cohort	Selection of the non-exposed cohort	Ascertainment of exposure	Demonstration that outcome of interest was not present at start of study	Study controls for most important factors	Study controls for other factors	Assessment of outcome	Was follow-up long enough for outcomes to occur	Adequacy of follow up of cohorts	Total Score (9 max)	Quality Rating	Overall Risk of Bias
Kohake et al. [12]	1	1	1	0	0	0	1	1	0	5	Moderate	Moderate
Verhage et al. [13]	1	1	1	0	1	0	1	1	0	6	Moderate	Moderate
Lambers et al. [14]	1	0	1	0	0	0	1	1	0	4	Low	High
Regan et al. [15]	1	0	1	0	0	0	1	1	0	4	Low	High
Ray et al. [17]	1	1	1	0	1	1	1	1	0	6	Moderate	Moderate
Chou et al. [16]	1	1	1	0	1	0	1	1	1	7	High	Low
Macera et al. [11]	1	0	1	0	0	0	1	0	1	4	Low	High
Axelrod et al. [10]	1	1	1	0	1	0	1	0	1	7	High	Low

### Surgical intervention secondary to post-traumatic osteoarthritis

The risk of surgical intervention for post-traumatic osteoarthritis ranged from 0% at 6 years in two studies [12, 13] to 4.2% at 17 months [17]. Kohake et al. (2019) reported no OA-related surgical interventions in their cohort of 61 patients (mean follow-up: 6.6 years) [12]. Similarly, Verhage et al. (2019) observed no OA-related re-operations in their study of 169 patients (mean follow-up: 6.3 years) [11, 13]. In the 50-patient cohort studied by Lambers et al. (2019) (mean follow-up: 21 years), one patient underwent ankle fusion 25 years post-injury, and another at 32 years, equating to 4% (n = 2) [14]. Regan et al. (2017) followed 141 patients (mean: 11.6 years) and reported 1 fusion and 1 replacement (0.7% each) [15]. In the 120-patient study by Ray et al. (2017) (mean follow-up: 17 months), 5 patients (4.2%) required ankle fusion [17]. Chou et al. (2023) observed 1 case of fusion (3.7%) in the ORIF group (n = 27) of their 49-patient cohort (mean follow-up: 46.6 months ORIF, 36.4 months AARMIS) [16]. Macera et al. highlighted that in a 378-patient cohort (follow-up: 24 months), 2.1% (n = 8) of patients required ankle fusion for advanced post-traumatic osteoarthritis [11]. Finally, Axelrod et al. (2020) analysed 44,133 operative cases (minimum follow-up: 24 months) and reported 0.5% (n≈221) undergoing ankle fusion and 0.2% (n≈88) ankle arthroplasty, using these as surrogates for end-stage osteoarthritis [10].

### Complications

Details of complications relating to the primary surgery for each study are summarised in Table 5. Mechanical issues such as broken or loose screws were reported in 3% of cases (n = 2) by Kohake et al. [12], while non-union and malunion occurred in 1.4% (n = 2) and 2.4% (n = 9) of patients, respectively in Regan et al. and Macera et al. [11, 15]. Wound complications were also notable, with infections reported in up to 6.5% (n = 4) of cases by Kohake et al. alongside wound necrosis and deep infection in 3.4% (n = 13) of the Macera et al. study [11, 12]. Radiographic evidence of post-traumatic osteoarthritis was frequently reported, ranging from 11% (n = 13) in Ray et al. to 63% (n = 89) [11, 13–15, 17], with 49% (n = 25) meeting van Dijk’s radiographic in the Lambers et al. study [14]. Implant-related issues led to hardware removal in 11% (n = 16) of patients [15].

**Table 5:** Outcomes of the included studies

References	Follow- up duration	Complications	Surgical intervention for OA	Time from injury	Further surgeries
Kohake et al. [12]	Mean: 6.6 years (range 2–12 years)	3% broken and 3% loose screws; 6.5% wound infections; 10% healing issues	0%	Not reported	32.7% syndesmotic screw removed after a minimum of six weeks postoperatively, with 3.2% concomitant removal of all hardware Further 14.7% of patients underwent removal of all hardware later point. 47.5% of patients with intact syndesmosis underwent implant removal
Verhage et al. [13]	Mean: 6.3 years (range 2.4–15.9 years)	Persistent step-off > 1 mm in 39%; osteoarthritis in 30%	0%	Not reported	No OA-related re-operations during follow-up, no information about other reoperation
Lambers et al. [14]	Mean: 21 years	49% radiographic signs of osteoarthritis according to the criteria of van Dijk	4% fusion	25 and 32 years	8% required additional surgery due to persistent pain or limited function. 2% hardware removal. 2% hardware removal subsequently developed a superficial wound infection, treated with oral antibiotics. 2% had an anteromedial arthrotomy to remove osteophytes, but the procedure was unsuccessful, and the pain persisted
Regan et al. [15]	Mean: 11.6 years (range 8.4–14.6 years)	11% underwent implant removal, 1.4% non-unions 63% evidence of post-traumatic osteoarthritis	0.7% fusion, 0.7% replacement	Not reported	11% implant removal
Ray et al. [17]	Mean: 17 months (range 2–100 months)	5.8% malreduction 5% infection 0.8% foot drop 0.8% synostosis 11% had osteoarthritis	4.2% fusion	Mean of 45 months	6.7% required revision surgery (5.8% malalignment, 0.8%infection), 37.5% screw removal
Chou et al. [16]	Mean ORIF: 46.6 ± 24.6 months Mean AARMIS: 36.4 ± 18.5 months	ORIF: 22.6% AARMIS: 13.6% Including wound necrosis, hardware irritation, sural paraesthesia, loss of reduction	3.7% of ORIF group fusion, 0% AARMIS	10 months	No information about other further surgeries, other than for osteoarthritis
Macera et al. [11]	24 months	Major complications were found in 31.5%: 17.2% residual pain, 3.4% deep infection, 2.4% malunion, 5% advanced posttraumatic ankle osteoarthritis, 0.3% implant breakage, 1.3% complex regional pain syndrome, 1.9% arthrofibrosis	2.1% fusion	Not reported	21.7% required reoperation: 4.5% open debridement of all necrotic and fibrous tissue and hardware removal was indicated for malunions, infections, extra-articular impingement, and implant breakage. 15.1% arthroscopic debridement for residual pain, intra articular impingement, arthrofibrosis, and early posttraumatic ankle OA
Axelrod et al. [10]	Minimum 24 months	No further complications recorded by this study	0.5% fusion, 0.2% replacement	Not reported	No information about other further surgeries, other than for osteoarthritis

### Other surgical interventions

The need for other additional surgery following the index ankle fracture varied among studies. Kohake et al. [12] reported that 20 patients underwent syndesmotic screw removal at a minimum of six weeks postoperatively, with 2 undergoing concomitant hardware removal and an additional 9 patients later having all hardware removed. Lambers et al. [14] reported 4 patients requiring further surgery due to persistent pain or limited function; three underwent additional surgical intervention including hardware removal and osteophyte excision, with the two mentioned above ultimately required ankle fusion. Regan et al. [15] reported 11% of patients needing implant removal. Ray et al. [17] observed that 6.7% of patients required revision surgery, due to malalignment or infection. Chou et al. [16] did not report further surgeries other than the fusion. Macera et al. [11] reported that in a 378-patient cohort with follow-up at 24 months, 15.1% (n = 57) underwent arthroscopic debridement for symptoms including early OA. Axelrod et al. [10] and Verhage et al. [13] did not report whether other surgical intervention were needed, other than for osteoarthritis.

## Discussion

Despite ankle fractures being common [1] and their recognised link to post-traumatic osteoarthritis [5, 6], data regarding the risk of secondary surgeries for arthritis are lacking [10]. Given the heterogeneity in study size, fracture severity, treatment modality and follow-up duration, a meta-analysis was deemed inappropriate. A narrative synthesis of the evidence was therefore performed, showing a risk of fusion or ankle replacement ranging from 0 to 4.2%. The results are predominantly driven by the Axelrod et al. data, reducing generalisability [10].

Kohake et al., Verhage et al. and Axelrod et al. reported 0 to 0.5% risk of fusion or replacement at follow-up durations of 6.3 to 6.6 years and 24 months, respectively [10, 12, 13] while Lambers et al. reported a fusion rate of only 4% with a mean follow-up of 21 years [14]. Although the average follow-up time across all included studies was approximately 6.8 years, this value was not weighted by study size, and many studies reported only minimum thresholds for follow-up, not exact follow-up times. As such, precise risk stratification over time and cumulative incidence cannot be calculated with the available data.

Previous literature regarding the development of ankle osteoarthritis post-surgical fixation of ankle fractures focuses upon radiographic indicators [18] with up to 63% of patients demonstrating radiographic signs of osteoarthritis [15]. In keeping with this literature, radiographic post-traumatic osteoarthritis was reported in five studies in our review, with prevalence ranging from 11 to 63% [11, 13–15, 17]. Despite this high prevalence, the rate of subsequent surgery for arthritis was low.

This discrepancy likely reflects both the poor correlation between radiographic degeneration and symptoms, and the relatively short follow-up durations of most studies. This poor correlation has been noted in other joints of the lower limb [19, 20], including the ankle [21]. Follow-up duration is a key determinant, as while some patients may already show evidence of secondary osteoarthritis early on, they are unlikely to have progressed to arthroplasty or fusion in that time frame, leaving the question of how many will ultimately require further surgical intervention unanswered.

Other surgical interventions were more frequently reported. Implant removal occurred in 10% of patients in two studies [14, 15] while Kohake et al. [12] reported syndesmotic screw removal in nearly one-third of cases. These findings are consistent with a large cohort study by Naumann et al. (2016), which reported an overall implant removal rate of 17%, including 14.4% due to subjective symptoms and 2.6% due to infection (18). A broader literature review by the same authors suggested implant removal rates ranging from 26 to 80%, consistent with the figures observed in this review [22].

Arthroscopic debridement was reported by Macera et al. [11], who found that 15.1% of patients had this intervention within 24 months of their initial surgery. Although arthroscopy may provide symptomatic relief in selected patients without advanced joint space narrowing, its long-term efficacy is unclear. Outcomes typically deteriorate with age, extent of cartilage damage, and radiographic disease severity [23]. Moreover, the rationale for arthroscopy in the Macera study was not clearly stated, suggesting potential variation in clinical practice.

### Limitations

Most of the cohort studies included in this review had small sample sizes. Given the high incidence of ankle fractures, the findings of these studies are not broadly generalisable. The exception was the registry study by Axelrod et al., which included 44,133 ankle fixations [10], comprising > 99% of the included patients, and disproportionately influencing the apparent incidence. This study reported follow-up to 20 years, during which patients undergoing operative and non-operative management were compared to matched individuals without ankle fractures. Approximately two-thirds of this cohort were managed non-operatively, substantially limiting the applicability of these findings to patients undergoing surgical fixation. Additionally, the process of matching lacks clarity and while large-scale registry studies such as Axelrod et al. [10] offer value in terms of large sample size and period of follow-up, their findings may be limited by the accuracy of coding for indication.

There was also considerable heterogeneity across the included studies in terms of fracture classification, mechanism of injury, imaging modalities, treatment approaches, follow-up durations, and outcome reporting. This variability limited the comparability of findings across studies and precluded the performance of meta-analysis. Furthermore, there was a lack of standardised diagnostic criteria for surgical intervention in post-traumatic ankle osteoarthritis.

Key variables such as body mass index (BMI), comorbidities, and baseline functional status were inconsistently reported. Ethnicity was not documented in any study. These omissions limit the ability to assess risk factors for developing post-traumatic osteoarthritis and to stratify outcomes by patient characteristics. None of the included studies stratified outcomes by fracture severity despite these factors being likely drivers of post-traumatic osteoarthritis and secondary ankle fusion or replacement surgery. Finally, the included studies often lacked clarity regarding complications which followed the index ankle fracture fixation. Therefore, it is difficult to determine whether the osteoarthritis which developed after the ankle fracture fixation was a direct result of the injury and its initial treatment or related to later complications such as deep surgical site infection or septic arthritis. This also relates to the variable duration of follow-up as it has been shown that complications of the index surgery led to a shorter latency time to development of end-stage osteoarthritis [24].

The time interval until onset of severe post-traumatic osteoarthritis of the ankle has been quoted to be 20 years [24]. Only one of the studies included in this review [14] followed the patients up for this timespan. The remaining studies had shorter follow-up periods, including a study with a mean follow-up of 17 months [17]. Therefore, there may be an underestimation of the long-term burden of surgery secondary to traumatic osteoarthritis, particularly in younger populations.

## Conclusion

The literature indicates that the risk of ankle fusion or replacement for ankle arthritis after an ankle fracture fixation is low within the first decade after injury; however, this conclusion is driven almost entirely by a single registry study and limited-duration cohort studies. This lack of evidence is particularly significant for young patients who might develop osteoarthritis many years after the injury. The true long-term risk of secondary surgery after ankle fracture fixation remains unknown. Further studies with clear definitions of osteoarthritis-related further surgeries are needed to better understand the burden of disease and to inform preventative strategies.

## Supplementary Information

Below is the link to the electronic supplementary material.

Supplementary Material 1.

## Data Availability

The data that support the findings of this study are available from the corresponding author upon reasonable request.
