# Supplementary material for: Likelihood of secondary surgery due to osteoarthritis following ankle fracture fixation: a systematic review
Source: J Orthop Surg Res. 2026 May 13;21:454. doi: 10.1186/s13018-026-06928-8 (PMC13430694; doi:10.1186/s13018-026-06928-8)
Supplement: Supplementary file 1 — Supplementary Material 1. [file 13018_2026_6928_MOESM1_ESM.docx]

**Appendix 1 – search strategy**

(exp ankle fractures/ or exp ankle injuries/ or ((ankle or malleolar or talus) adj3 (injur* or

trauma or fracture*)).ti,ab,kf.) and (exp osteoarthritis/ or arthritis, osteoarthritis/ or

arthriti*.ti,ab,kf. or degenerative joint disease.ti,ab,kf. or osteoarthros*.ti,ab,kf.) and (exp

surgical procedures, operative/ or surg*.ti,ab,kf.)
